# Automatic Tuning Method for Quadrupole Mass Spectrometer Based on Improved Differential Evolution Algorithm

**DOI:** 10.3390/bioengineering12111154

**Published:** 2025-10-24

**Authors:** Yuanqing Zhang, Baolin Xiong, Le Feng, Liang Li, Wenbo Cheng, Yuguo Tang

**Affiliations:** 1 School of Biomedical Engineering (Suzhou), Division of Life Sciences and Medicine, University of Science and Technology of China, Hefei 230026, China; zhangyq@sibet.ac.cn (Y.Z.);; 2 Suzhou Institute of Biomedical Engineering and Technology, Chinese Academy of Sciences, Suzhou 215163, China; 3 Tianjin Key Laboratory of Medical Mass Spectrometry for Accurate Diagnosis, Tianjin 300399, China

**Keywords:** quadrupole mass spectrometer, automatic tuning method, improved differential evolution algorithm, subpopulation, mutation strategy

## Abstract

Quadrupole mass spectrometers are highly sensitive and specific analytical instruments, widely used in pharmaceuticals, clinical diagnostics, and other fields. Their performance depends on a tuning process to optimize key parameters, which has traditionally relied on engineers’ expertise or simple univariate search methods. This paper proposes an automatic tuning method using an improved differential evolution algorithm. This algorithm introduces a ranking and subpopulation classification for individuals, enabling distinct mutation strategies. Validation on the CEC-2017 benchmark functions confirms the superiority of the improved algorithm. In automatic tuning experiments, it achieved a 25.3% performance gain over the univariate search method and also surpassed both the classical differential evolution algorithm and standard particle swarm optimization algorithm. This method proves to be an effective approach for enhancing the performance of quadrupole mass spectrometers.

## 1. Introduction

Quadrupole mass spectrometers are advanced and sophisticated analytical instruments, recognized for their high sensitivity and strong specificity. They are widely used in fields such as pharmaceuticals, food safety, and clinical diagnosis [1,2,3,4,5,6]. Quadrupole mass spectrometers are relatively complex devices. Instrument tuning is involved in the production, use, and after-sales service. This tuning process may encompass ten or more parameters. The traditional tuning method typically uses univariate search, i.e., optimizing each parameter individually and accepting the resulting set of parameters as optimal. However, the univariate search method does not account for the interactions among parameters, and the result might not be optimized sufficiently. Therefore, it is essential to develop an efficient multi-parameter optimization algorithm.

Most real-world tuning problems are complex, large-scale, and involve nonlinearity and constraints. The search processes of traditional algorithms often struggle to adequately explore the vast parameter space, failing to locate the global optimum or a satisfactory near-optimal result. Inspired by natural phenomena, numerous intelligent optimization algorithms have been developed, including: (1) genetic algorithms and immune algorithms based on the physiological and evolutionary mechanisms; (2) swarm intelligence algorithms based on the competition and cooperation found among natural populations, such as the particle swarm optimization algorithms and ant colony algorithms; (3) simulated annealing algorithms inspired by physical phenomena; (4) other artificial intelligence algorithms such as the artificial neural network algorithms.

The differential evolution (DE) algorithm was proposed by Storn in 1995 to solve the Chebyshev polynomial problem [7]. The DE algorithm is classified as a population-based algorithm (nature-inspired algorithm). The performance of DE was showcased at the First International Contest on Evolutionary Optimization in May 1996. Since then, it has become one of the most popular optimizers for complex optimization problems [8,9,10,11,12]. However, the mutation strategy can sometimes lead to poor performance in terms of diversity of search directions and convergence speed, resulting in low optimization efficiency. Liu and Lampinen introduced the fuzzy adaptive differential evolution (FADE), which employs fuzzy logic controllers to adapt the control parameters F and CR for the mutation and crossover operations, demonstrating better results than the classic DE when problem dimensions are high [13,14,15,16]. To enhance population diversity and improve convergence performance, Zhang and Sanderson proposed an adaptive differential evolution (JADE) [17]. This approach implemented a mutation strategy called “DE/current-to-pbest” with an optional archive and adaptively controlled F and CR. Tests on traditional benchmark functions showed competitive performance regarding convergence rate and reliability compared to other classic and adaptive DE algorithms and the PSO algorithm. Based on the SHADE algorithm, Tanabe and Fukunaga introduced the L-SHADE algorithm, which further extends SHADE with Linear Population Size Reduction (LPSR) and continuously reduces the population size according to a linear function [18,19,20,21,22,23,24,25,26]. Several improved DE algorithms based on L-SHADE performed well in the CEC competition [27,28,29,30]. Duankhan proposed the differentiated creative search (DCS) algorithm, which is similar to the DE algorithm [31]. It utilizes a newly proposed dual-strategy approach that balances divergent and convergent thinking within a team-based framework, along with a unique knowledge-acquisition process.

The particle swarm optimization (PSO) algorithm is another optimization algorithm that exhibits excellent performance. It was proposed in 1995 by James Kennedy and Russell Eberhart, who were inspired by the foraging behavior of birds [32]. Shi introduced the inertia weight into the original particle swarm optimizer [33]. A time decreasing inertia weight can bring in a significant improvement on the PSO performance. This improved PSO algorithm is referred to as the standard PSO algorithm. In most cases, it is recommended that the inertia weight of the standard PSO algorithm linearly decreases from 0.9 to 0.4.

For the automatic tuning of mass spectrometers, existing commercial instruments typically offer simple automatic tuning programs based on the univariate search method. For instance, both the Thermo Scientific iCAP TQ ICP-MS and the SCIEX Triple Quad 5500 system have implemented this method. Additionally, considering the user’s acceptance, the automatic tuning time is usually limited to about three hours. This requires that automatic tuning methods be quick and efficient. Dawson provided a detailed exposition of the stability diagram and mass resolution calculations for quadrupole mass analyzers [34]. The mass resolution in a quadrupole mass spectrometer is usually defined as the full width at half maximum (FWHM). Syed [35] and Liu [36] respectively presented simulation cases and experimental cases of mass resolution adjustment. Automatic tuning of peak intensity is not mentioned in these papers. Zhang utilized the genetic algorithm to perform adaptive optimization tests on voltage parameters for the self-developed MR-TOFMS instrument [37]. Compared to the step-scan method, the adaptive optimization approach completed the global search for five voltage parameters in 2.25 h. The sensitivity and resolution of the mass spectrometer improved by 52.4% and 50.6%, respectively, compared to the step-scan optimization method. However, for general mass spectrometers with numerous parameters, further improvements in algorithm efficiency are necessary. Jia proposed an automatic tuning method for mass spectrometers based on an improved particle swarm optimization algorithm [38]. This method employs four calculation methods for the inertia weight: linear weight, exponential weight, power weight, and random weight, to obtain fitness values separately. It yielded good results, but compared to the single inertia weight method, it incurred higher time costs.

Given the challenges in modeling and the limited optimization time during the tuning process of mass spectrometers, a method utilizing an improved differential evolution algorithm to tackle the multi-parameter optimization is proposed. Compared to traditional manual tuning and the automatic tuning method based on univariate search, it can produce superior optimization results. Regarding the commercial instruments and software, there are two approaches to implement the application of the algorithm. The first approach is similar to the method employed in this paper. The algorithm is an independent program. The algorithm and instrument communicate via text files to exchange tuning parameters and peak intensity results. However, this requires the instrument software to permit manipulation through parameterized script files and the execution of the tuning process. The second approach involves the instrument manufacturer modifying the instrument software and integrating the algorithm into it.

## 2. Methods

The differential evolution (DE) algorithm is a random heuristic search algorithm. Its operational steps include initialization, mutation, crossover, boundary condition handling, and selection.

The main ideas of the improved differential evolution algorithm are: (1) introducing a factor related to the number of iteration to allow the algorithm to exhibit adaptive search ranges and speeds during the iteration process; (2) applying different mutation strategies based on the performance of the population; (3) ranking the individuals according to the fitness values to guide the next iteration; (4) incorporating a random search strategy to prevent the algorithm from getting stuck in a locally optimal result.

In this paper, three subpopulations are defined: the elite subpopulation (ELS), the middle subpopulation (MIS), and the inferior subpopulation (INS). In the classic DE algorithm, the mutation factor is conventionally considered a constant. Throughout the iterative process, the value of this factor markedly influences the outcomes. A mutation factor that is excessively large may lead the algorithm to perform a random search, thereby reducing search efficiency. Conversely, a mutation factor that is too small can diminish population diversity, increasing the risk of “premature convergence,” which refers to the algorithm becoming trapped in a local optimal result. Consequently, it is advisable to incorporate adaptive mutation factors within the mutation operation process.

**Figure 1:**
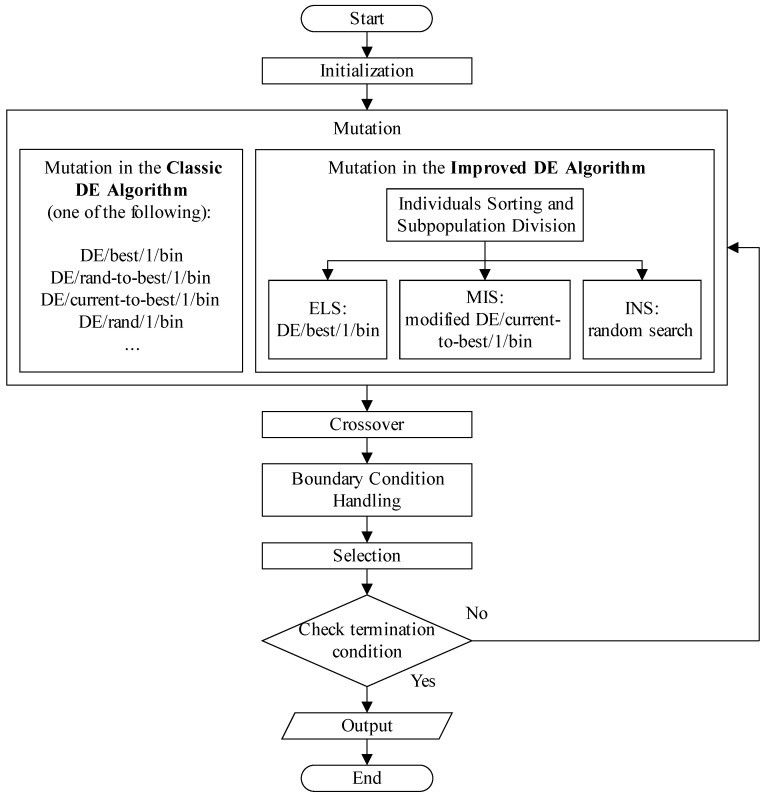
Flowchart of DE algorithm (the classic DE algorithm and the improved DE algorithm).

The flowchart of DE algorithm (both the classic DE algorithm and the improved DE algorithm) is shown in Figure 1.

### 2.1. Differential Evolution Algorithm

The population of the DE algorithm consists of *N* individuals. Each individual is a vector and can be described as ***x****_i_* = (*x*_1,*i*_, *x*_2,*i*_, …, *x_D_*_,*i*_,), *i* = 1, 2, …, *N*, where *D* is the dimension of the vector and *N* is the population size. The formula for calculating the mutation vector in the classic differential evolution algorithm is:

***v***_*i*,*g*__+1_ = ***x****_r_*_1,*g*_ + *F* × (***x****_r_*_2,*g*_ − ***x****_r_*_3,*g*_) (1)

Here, *r*1, *r*2, *r*3 are integers randomly selected within the range [1, *N*], and *r*1 ≠ *r*2 ≠ *r*3 ≠ *i*. The mutation factor *F* is a real constant within the range [0, 2]. *g* and *g* + 1 present the number of iterations.

The mutation strategy includes several other forms, identified by the symbols DE/x/y/z. Here, x determines whether the current mutation vector is random, optimal, or current; y signifies the number of differential vectors utilized; and z indicates the operational strategy during the crossover step, which can include options such as linear or exponential. Other forms of mutation strategies include:

(a) DE/best/1/bin:

***v***_*i*,*g*__+1_ = ***x***_*best*,*g*_ + *F* × (***x****_r_*_1,*g*_ − ***x****_r_*_2,*g*_) (2)

(b) DE/rand-to-best/1/bin:

***v***_*i*,*g*__+1_ = ***x***_*i*,*g*_ + λ × (***x****_best,g_* − ***x***_*i*,*g*_) + *F* × (***x****_r_*_1,*g*_ − ***x****_r_*_2,*g*_) (3)

The steps of the DE algorithm are as follow Algorithm 1:
**Algorithm 1.** Differential Evolution AlgorithmInput: Set the population size *N*, and the boundaries ***x****^ub^* and ***x****^lb^*. Step 1 (Initialization): Randomly generate the population and obtain the corresponding fitness values.*x_j,i,_*_0_ = *x^lb^_j_ +* rand[0,1] × (*x^ub^_j_* − *x^lb^_j_*)(4)Here, rand [0,1] represents the uniformly generated random number within the range [0,1]. Step 2 (Mutation): A mutant vector is generated according to the mutation formula. Step 3 (Crossover): A trial vector is generated in crossover between the target vector and mutant vector using a crossover probability *CR*. If randb(*j*) ≤ *CR* or *j* = rnbr(*i*), then *u_j,i,g_*_+1_ = *v_j,i,g_*_+1_; if randb(*j*) > *CR* and *j* ≠ rnbr(*i*), then *u_j,i,g_*_+1_ = *x_j,i,g_*_+1_, (*j* = 1,2,…,*D*); Here, randb(*j*) represents the *j*-th value of the random number sequence generated within the range of [0,1]; *CR* represents the crossover operator, whose range of values is [0,1]; rnbr(*i*) represents the random number sequence generated from [1, *D*], thereby achieving the acquisition of at least one parameter value from the parameter vector to the mutated parameter vector, and thereby achieving the update of the parameter vector rather than keeping it unchanged. Step 4 (Boundary Condition Handling): After the crossover operation, check whether the population exceeds the position boundaries. If it does, set it to the relevant position boundaries. If *u_j,i,g_*_+1_ > *x^ub^_j_*, then *u_j,i,g_*_+1_ = *x^ub^_j_*; If *u_j,i,g_*_+1_ < *x^lb^_j_*, then *u_j,i,g_*_+1_ = *x^lb^_j_*. Here, *x^lb^_j_* and *x^ub^_j_* are the lower and upper limits of the boundaries respectively, and j is the j-th dimension of the vector. Step 5 (Selection): Calculate the fitness values and perform the selection operation. If f(***u****_i,g_*_+1_) < f(***x****_i,g_*), ***x****_i,g+_*_1_ = ***u****_i,g_*_+1_; If f(***u****_i,g_*_+1_) < f(***x****_i,g_*), ***x****_i,g+_*_1_ = ***x****_i,g_* Step 6: Check whether the iteration termination condition is met. If it is, terminate the iteration. If not, return to Step 2. Output: The global optimal vector and fitness value.


### 2.2. Improved Differential Evolution Algorithm

The operational steps of the improved DE algorithm are the same as those of the classic DE algorithm. The primary difference between the improved DE algorithm and the classical DE algorithm lies in the mutation step.

In the mutation step, sort the individuals in ascending order by their fitness values to create a new population. Divide the new population into subpopulations based on the distinct performances of individuals to apply different mutation operations. Additionally, the size of the subpopulations can be adjusted throughout the iteration process.

The classification of subpopulations is as follows: in the initial stage of the iteration process (g ≤ G/3), the ratio of ELS: MIS: INS is 2:5:3; in the middle stage of the iteration process (G/3 < g ≤ 2G/3), the ratio is 4:4:2; and in the final stage of the iteration process (g > 2G/3), the ratio is 6:3:1.

In the early stages of the iteration process, a large mutation factor is maintained to preserve the diversity of individuals and prevent premature convergence. In the later stages of the iteration process, the mutation operator gradually decreases, guiding the algorithm to converge to the optimal result.

Different mutation operations are subsequently adopted for various subpopulations.

(1) For the elite subpopulation, the search occurs close to the current global optimal position, and the DE/best/1/bin strategy is applied. Unlike the velocity of the particle swarm optimization, which is related to the distance from the optimal result, here it does not depend on the distance to the optimal point. The search range can be gradually diminished as the mutation factor F1 decreases.

***v***_*i,g*+1_ = ***x**_best,g_* + *F*_1_ ×
(***x**_r_*_1,*g*_ −
***x**_r_*_2,*g*_) (5)

*F*_1,*g*_ = *α*_1_ × rand[0,1] × [(*g*/*G*)^2^ − 2*g*/*G +* 1] (6)

(2) For the middle subpopulation, based on its current position, it considers moving toward the global optimal position while also accounting for random search, and executes the modified DE/current-to-best/1/bin strategy. The size of the middle subpopulation will gradually decrease as the iteration process.

***v****_i,g_*_+1_ = ***x****_i,g_* + *F*_2_ × (***x****_best,g_* − ***x***_i,*g*_) + *F*_3_ × (***x****_r_*_1,*g*_ − ***x****_r_*_2,*g*_) (7)

*F*_3*,g*_ = *α*_2_ *+ α*_3_ × [1 − sqrt(*g*/*G*)] (8)

Here, *F*_2_ is a real constant.

(3) For the inferior subpopulation, a random search strategy is employed, and the individuals are randomly generated within the feasible region. This subpopulation will gradually decrease in size during the iteration process.

*v_j,i,g_*_+1_ = *x^lb^_j_ +* rand[0,1] × (*x^ub^_j_* − *x^lb^_j_*) (9)

The parameters in the improved DE algorithm are defined as follows: α1 = 1; F2 = 0.4; α2 = 0.3; α3 = 0.3; CR = 0.75.

### 2.3. Automatic Tuning Method Based on the Improved Differential Evolution Algorithm

The automatic tuning of a triple quadrupole mass spectrometer involves parameters related to the gas in the ion source and interface, as well as the voltage applied to the ion optical lenses. The goal of tuning and optimization is to achieve the best peak intensity of the mass spectrum while ensuring that the accuracy of the mass axis and resolution meet specific requirements.

A commercial mass spectrometer (LC-HTQ 2030 LC-MS/MS, Tianjin Guoke Medical Science and Technology Development Co., Ltd., Tianjin, China) is selected as the test platform. The software used to drive the instrument is the LCMSMS OS V1.0. The test samples consist of a mixed solution of NaI and CsI (NaI 200 μg/mL and CsI 5 μg/mL).

**Figure 2:**
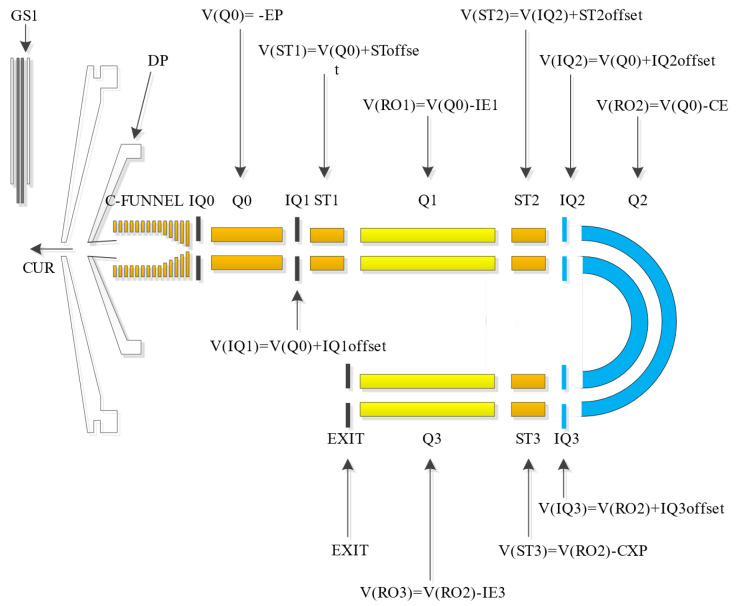
Schematic diagram of the ion source and lens parameters of the triple quadrupole mass spectrometer.

**Figure 3:**
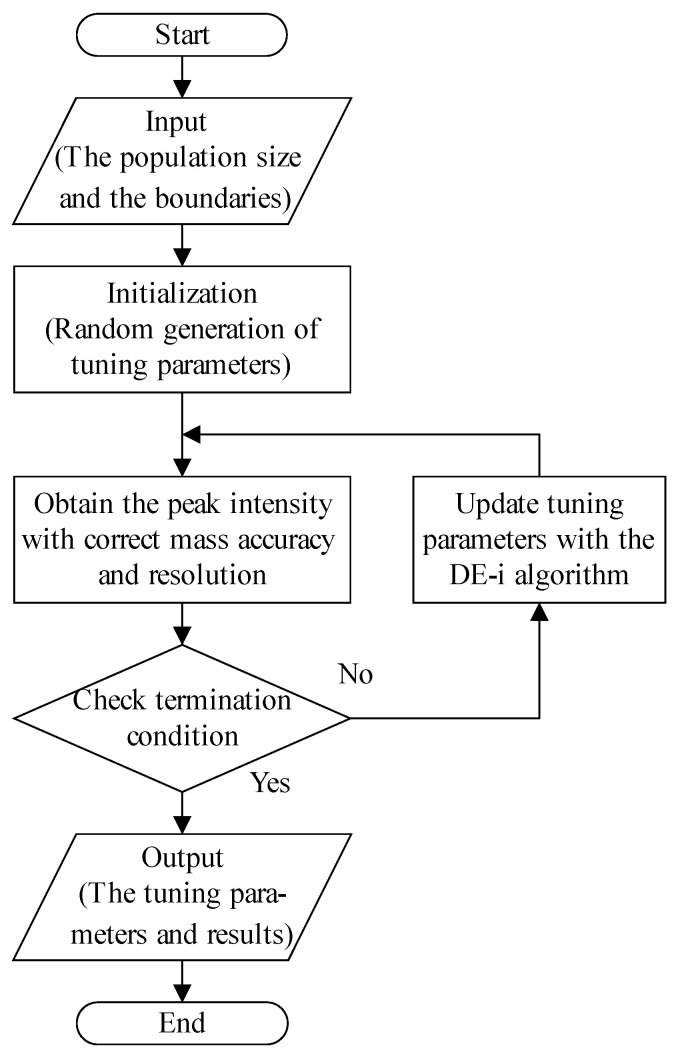
Flowchart of the automatic tuning method based on the improved differential evolution algorithm.

Figure 2 illustrates a schematic diagram of the ion source and ion optics lens parameters of the test platform. The flowchart of the automatic tuning method based on the improved differential evolution algorithm is shown in Figure 3. The algorithm and instrument communicate via text files to exchange tuning parameters and peak intensity results. Operation on the mass spectrometer encompasses calibration of the mass axis and mass resolution, and peak intensity result output. The calibration of the mass axis and resolution is relatively simple and can be achieved through a linear model with theoretical formulas.

**Figure 4:**
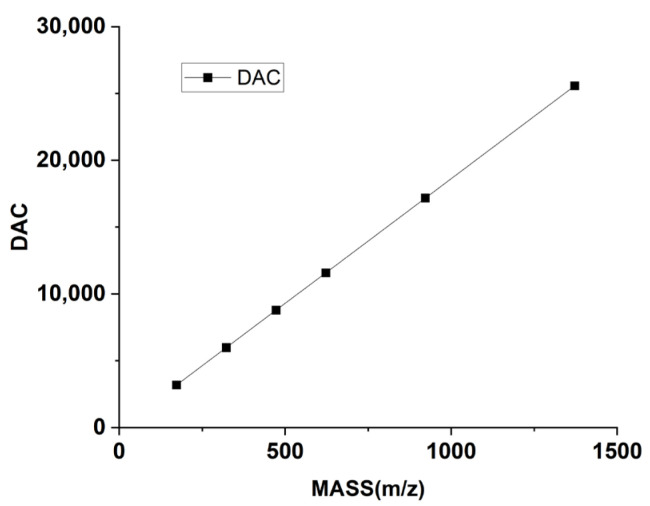
Correspondence of mass to DAC.

**Figure 5:**
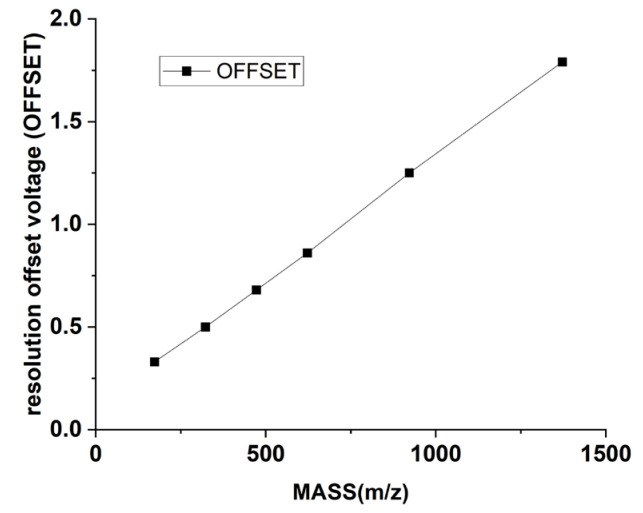
Correspondence of mass to resolution offset voltage (OFFSET).

Taking the Q1 mode as an example, the primary parameters involved in the tuning process include gas parameters such as the nebulizer gas (GS1) and curtain gas (CUR), along with voltage parameters like de-clustering voltage (DP), entrance voltage (EP). Parameters like GS1 and CUR, as well as DP and EP, primarily influence the intensity of the mass spectral peak. The mass axis is determined by a series of mass numbers and digital-analog converter (DAC) values that are in a linear relationship. The DAC value corresponds to the magnitudes of both direct voltage and radio-frequency voltage at a specified mass number. DAC impacts the accuracy of the mass axis. The resolution offset voltage (OFFSET) affects the mass resolution. The instrument is factory-set with values of DAC and OFFSET corresponding to a series of mass numbers, which are shown in Figure 4 and Figure 5. Figure 4 illustrates the relationship between mass and DAC for the instrument, and Figure 5 illustrates the relationship between mass and OFFSET.

The linear model for calibration of the mass axis with the voltage DAC is

*INT_DAC_m_* = *INT_DAC_m_*_−1_
*+ k* × (*mass*_target_ − *mass_m_*_−1_) (10)

Here, *INT_DAC_m_* and *INT_DAC_m-_*_1_ are the voltage values for the mass axis in the *m*-th and (*m* − 1)-th iteration; *k* is the coefficient; *mass*_target_ is the target value of the mass axis; *mass_m-_*_1_ is the mass axis value in the (*m* − 1)-th iteration.

The termination condition for the iteration is:

|*mass*_target_ − *mass_m_*| ≤ 0.1 (11)

Here, *mass_m_* is the current value of the mass axis in the *m*-th iteration.

The linear model for calibration of mass resolution with the voltage OFFSET is

*V_RES_m_* = *V_RES_m_*_−1_
*+ l* × (*res*_target_ − *res_m_*_−1_) (12)

Here, *V_RES_m_* and *V_RES_m_*_−1_ are the voltage values for the mass resolution in the *m*-th and (*m* − 1)-th iteration; *l* is the coefficient; *res*_target_ is the target value of the mass resolution; *res_m_*_−1_ is the mass resolution value in the (*m* − 1)-th iteration.

The termination condition for adjusting the mass resolution is:

|*res*_target_ − *res_m_*| ≤ 0.1 (13)

Here, *res_m_* is the value of the mass resolution in the *m*-th iteration. Typically, the target value of the mass resolution is set to 0.7.

After calibration of the mass axis and mass resolution, the peak intensity results are output to the algorithm.

A multi-objective evaluation function is required to optimize the intensities of several mass spectral peaks. A function for multi-objective evaluation is defined as follows:

*f*(***x***) = *λ*_1_/*I*_1_ + *λ*_2_/*I*_2_ + *λ*_3_/*I*_3_ (14)

Here, ***x*** is a vector composed of the ion source and ion lens parameters. *I*_1_, *I*_2_, and *I*_3_ represent the intensities of the typical mass spectral peaks in the low, medium, and high mass number. *λ*_1_, *λ*_2_, and *λ*_3_ represent the weight coefficients of three mass spectral peaks.

In the subsequent trials, *λ*_1_ = *λ*_2_ = *λ*_3_ = 10^6^. The improved DE algorithm computes fitness values through the evaluation function and generates the new tuning parameter file according to the internal rules of the algorithm.

After the automatic tuning process, the tuning results including the optimized parameter values and the intensities of the mass spectral peaks are output. The calibration of the mass axis and mass resolution are implicitly carried out during the execution of the automatic tuning method.

It’s critical to note that the automatic tuning time of the mass spectrometer is typically kept within three hours. For example, during the acquisition of mass spectral data, the scanning time for each mass spectral peak is set to 0.6 s. When there are three mass spectral peaks and MCA (Multiple Channel Acquisition) is set to 5, the total time required is about 10 s. Therefore, about 360 mass spectral data can be obtained in one hour. Typically, the automatic tuning process can get 500 to 1000 mass spectral data entries.

## 3. Results and Discussion

### 3.1. Benchmark Function Test

The improved DE algorithm (DE-i) was compared with the classic DE algorithm and the standard particle swarm optimization algorithm (PSO). The classic DE algorithm used two mutation methods: DE/best/1/bin and DE/rand-to-best/1/bin.

To evaluate the optimization algorithms, the IEEE Congress on Evolutionary Computation (IEEE CEC) has provided a series of standardized benchmarks. The CEC-2017 test function set developed by Awad was used in this paper [39]. The test function set includes a variety of widely used and recognized functions, such as unimodal, multimodal, mixed, and combined functions. F1 and F3 are unimodal functions with a single global minimum and no local extrema. F4–F10 are multimodal functions with local extrema. F11–F20 are mixed functions that combine three or more benchmark functions. F21–F30 are composite functions made up of at least three mixed or benchmark functions. However, F2 is officially removed due to stability problems.

Considering that actual mass spectrometer test data exhibits certain random fluctuations, the test functions were selected from F4–F30 and are shown in Table 1.

Regarding the limited tuning time and the total iterations in the automatic tuning process, the population size is set to 20, and the maximum number of iterations is set to 25. With these constraints, the performance of the four optimization algorithms is evaluated. The dimension is set to 10. The evaluation of different optimization algorithms is based on statistical data from 30 repeated trials.

**Figure 6:**
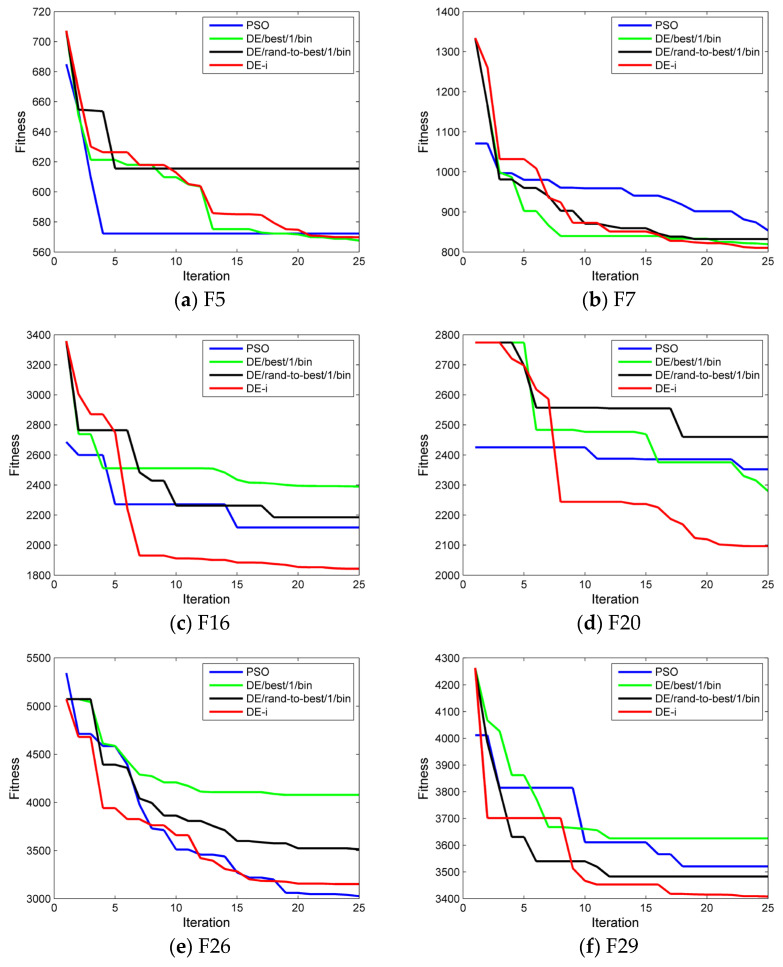
Some convergence curves of CEC-2017 functions for different algorithms among the 30 repeated trials.

After 30 repeated trials, the mean value of the function results obtained through optimization by a specific algorithm is employed to evaluate the optimization performance of the algorithm. A smaller mean value indicates that the algorithm has stronger optimization ability and is closer to the theoretical optimal result Fi* of the function. The standard deviation (std) is employed to assess the consistency of the results from 30 repeated trials, yet it is not directly utilized to evaluate the performance of algorithms. The statistical results of the test functions for different algorithms are shown in Table 2. Some convergence curves, selected from the 30 repeated trials, are shown in Figure 6.

As shown in Table 2, the improved DE algorithm exhibits superior performance in functions F5, F7, F16, and F20. The PSO algorithm demonstrates better performance in function F26. The classic DE algorithm employing the DE/best/1/bin mutation strategy shows superior performance in function F29. The results indicate that under limited optimization conditions, the improved DE algorithm outperforms the classic DE algorithm in optimization performance and provides certain advantages over the standard PSO algorithm. It is expected that the improved DE algorithm will achieve efficient optimization for mass spectrometers.

### 3.2. Application of Automatic Tuning Method

#### 3.2.1. Optimization of a Single Mass Spectral Peak

The automatic tuning method was used for the intensity optimization of a single mass spectral peak at *m*/*z* 172.88. Before tuning, the intensity of the *m*/*z* 172.88 mass spectral peak is 2.08 × 10^7^ cps. The mass accuracy and mass resolution are 0.103 u and 1.239 u. Parameters that required optimization include CUR, GS1, DP, and EP. Five methods were employed for tuning.

**Figure 7:**
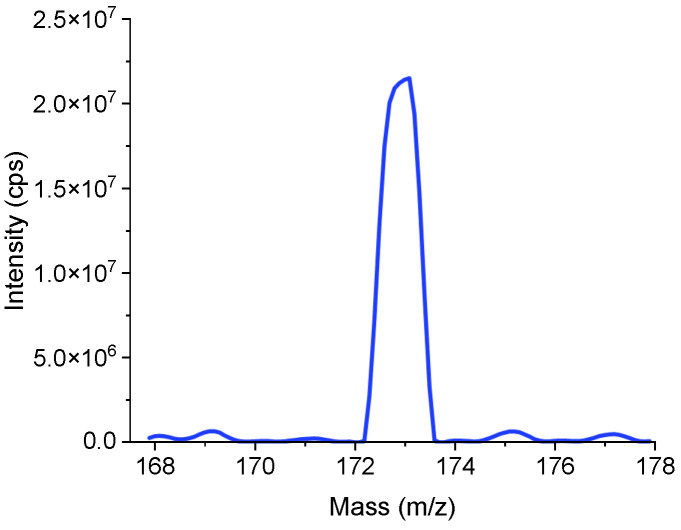
Mass spectrum obtained after univariate search (intensity: 2.15 × 10^7^ cps; mass resolution: 0.786 u).

The first method is univariate search. Ten uniformly distributed parameter values are selected within the feasible interval for each parameter. The process took 5 min to obtain the result, including the time for file reading and writing. Final optimal parameter values obtained were: GS1 =20; CUR = 10; DP = 120; EP = 10. The mass spectrum is displayed in Figure 7. The intensity of the *m*/*z* 172.88 mass spectral peak is 2.15 × 10^7^ cps. The mass resolution is 0.786 u. Here and in all subsequent mass spectrum, MCA = 5 is adopted.

The other four tuning methods employed the four optimization algorithms mentioned earlier. The population size is set to 20, and the maximum number of iterations is set to 25. The duration of tuning is primarily determined by the volume of generated mass spectral intensity data. The computational time required by these four intelligent optimization algorithms is largely comparable, all falling within the range of 0.8 to 1 h.

The evaluation function is shown in Equation (15).

*f*(***x***) = *λ*_1_/*I*_1_ (15)

**Figure 8:**
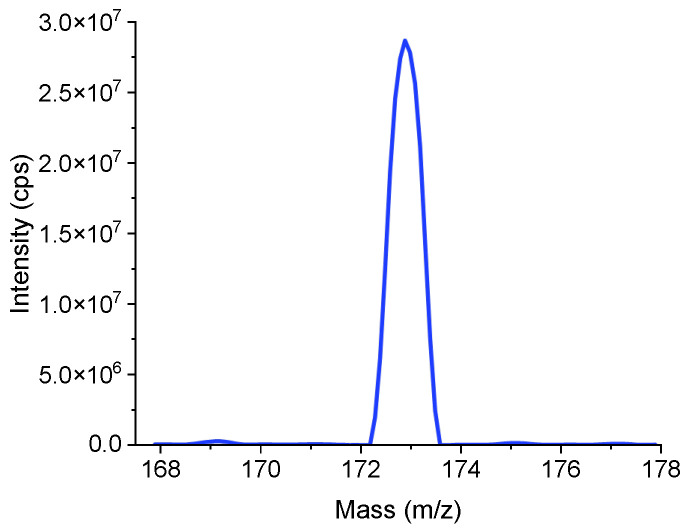
Mass spectrum obtained after the automatic tuning method based on the improved DE algorithm (intensity: 2.87 × 10^7^ cps; mass resolution: 0.675 u).

All of the results are shown in Table 3. The data from the automatic tuning process are shown as follows. The optimal values of the parameters obtained based on the improved DE algorithm were: CUR = 20.229; GS1 = 15.573; DP = 163.03; EP = 11.413. The peak intensity data were relatively close. The improved DE algorithm gives the best peak intensity data from other methods. The intensity obtained by this algorithm is 25.3% higher than that of the univariate search method. The final mass spectrum is shown in Figure 8. It should be noted that during the automatic tuning process, only intensities of the mass spectral peaks were output, without saving the mass spectrum files. The mass spectrum shown in Figure 8 was generated by re-running the mass spectrometer with the optimized parameters to produce a new mass spectrum file. Consequently, due to random variations between experimental replicates, minor discrepancies may exist between the intensities reported in Table 3 and those displayed in Figure 8. Subsequent mass spectra were generated and output in the same way. The differences do not affect the conclusion. The results indicate that the automatic tuning method based on the improved differential evolution algorithm can achieve better performance for the instrument.

#### 3.2.2. Automatic Tuning of the Ion Source and Lens Parameters

**Figure 9:**
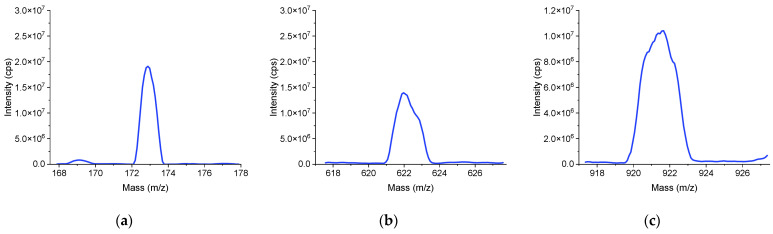
The mass spectrum before the tuning process. (**a**) *m*/*z* 172.88 (intensity: 1.91 × 10^7^ cps; mass resolution: 0.814 u). (**b**) *m*/*z* 622.57 (intensity: 1.39 × 10^7^ cps; mass resolution: 1.313 u). (**c**) *m*/*z* 922.36 (intensity: 1.04 × 10^7^ cps; mass resolution: 1.945 u).

**Figure 10:**
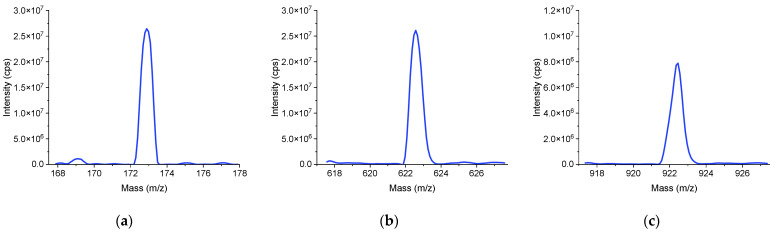
The mass spectrum after the tuning process. (**a**) *m*/*z* 172.88 (intensity: 2.64 × 10^7^ cps; mass resolution: 0.675 u). (**b**) *m*/*z* 622.57 (intensity: 2.61 × 10^7^ cps; mass resolution: 0.709 u). (**c**) *m*/*z* 922.36 (intensity: 7.89 × 10^6^ cps; mass resolution: 0.723 u).

Ten repeated trials of automatic tuning were conducted to verify its repeatability. The performance evaluation involves the intensities of three mass spectral peaks (*m*/*z* 172.88, 622.57, 922.36). The evaluation function is defined in Equation (14). The population size is set to 20, and the maximum number of iterations is set to 25. Conducting a single automatic tuning process involving three mass spectrometry peaks requires 2.5 h. This includes the time for storing the data files during the process. The test data are shown in Table 4. Each mass spectral peak met the requirements for mass accuracy and mass resolution. The CV values of the peak (*m*/*z* 172.88, 622.57, 922,36) intensities were 5.1%, 6.2%, and 9.6%, respectively. The results indicate that the method achieved calibration of mass accuracy and resolution, successfully completing the tuning of the instrument’s performance. Figure 9 presents the mass spectrum in its initial state, which displays a certain positional offset of the mass spectral peaks along with poor mass resolution and low peak intensities. Figure 10 shows the mass spectrum after applying the automatic tuning method. By comparing the data before and after automatic tuning, it is evident that mass accuracy, mass resolution, and peak intensity have all significantly improved.

For quadrupole mass spectrometers, the terminology employed by instruments from different manufacturers may vary, and design configurations can differ. However, the definitions and adjustment principles for mass accuracy and mass resolution in quadrupole mass analyzers are fundamentally consistent. The tuning parameters for ion sources and ion transmission systems commonly used in quadrupole mass spectrometers are also analogous. Consequently, it is reasonable to anticipate that an automatic tuning method possesses general applicability to quadrupole mass spectrometer systems. The improved DE algorithm, as an intelligent optimization algorithm, exhibits universality in multi-parameter optimization. Nevertheless, experimental validation is required to ascertain its applicability for automatic tuning of other mass spectrometer types.

## 4. Conclusions

The traditional process of automatic tuning in mass spectrometers typically optimizes each parameter individually and regards the results as the best values. This paper presents an automatic tuning method based on the improved differential evolution algorithm. Comparison tests of standard functions demonstrate that under specific conditions, the improved differential evolution algorithm significantly outperforms the traditional differential evolution algorithm and shows certain advantages over the standard particle swarm optimization algorithm. This algorithm has produced impressive optimization results in the actual tuning of tandem quadrupole mass spectrometers. The algorithm is scheduled to be employed in tuning experiments for other types of mass spectrometers, such as time-of-flight mass spectrometers, ion trap mass spectrometers, and various hybrid mass spectrometers, to evaluate its applicability. Additionally, the ideas within this algorithm are expected to inspire other researchers and improve the traditional optimization algorithms, such as the particle swarm optimization algorithm.

## Figures and Tables

**Table 1:** The CEC-2017 test functions used for performance comparison of different algorithms [39].

Functions Type	Functions No. of CEC 2017	Functions	Fi* = Fi(x*)
Simple Multimodal Functions	F5	Shifted and Rotated Rastrigin’s Function $f_{5}(x)=\sum_{i=1}^{D}\left(x_{i}^{2}-10cos(2\pi x_{i})+10\right)$	500
	F7	Shifted and Rotated Lunacek Bi_Rastrigin Function $f_{7}(x)=min(\sum_{i=1}^{D}{\left({\hat{x}}_{i}-\mu_{0}\right)}^{2},dD+s\sum_{i=1}^{D}{\left({\hat{x}}_{i}-\mu_{0}\right)}^{2})+10(D-\sum_{i=1}^{D}cos(2\pi {\hat{z}}_{i}))$	700
Hybrid Functions	F16	Hybrid Function 6 (*N* = 4) $F(x)=g_{1}(M_{1}z_{1})+g_{2}(M_{2}z_{2})+...+g_{N}(M_{N}z_{N})+F^{*}(x)$	1600
	F20	Hybrid Function 10 (*N* = 6) $F(x)=g_{1}(M_{1}z_{1})+g_{2}(M_{2}z_{2})+...+g_{N}(M_{N}z_{N})+F^{*}(x)$	2000
Composition Functions	F26	Composition Function 6 (*N* = 5) $F(x)=\sum_{i=1}^{N}\left\{\omega_{i}*[\lambda_{i}g_{i}(x)+{bias}_{i}]\right\}+F^{*}(x)$	2600
	F29	Composition Function 10 (*N* = 3) $F(x)=\sum_{i=1}^{N}\left\{\omega_{i}*[\lambda_{i}g_{i}(x)+{bias}_{i}]\right\}+F^{*}(x)$	2900

**Table 2:** Statistical results of the CEC-2017 test functions of 30 repeated trials for different algorithms. (D = 10, NP = 20, G = 25, the best result is marked in bold).

Functions	-	PSO	DE/Best/1/Bin	DE/Rand-to-Best/1/Bin	DE-i
F5	Mean	562.41	564.73	583.78	**553.74**
	Std	10.25	27.62	19.14	19.55
F7	Mean	819.14	844.19	834.90	**803.13**
	Std	18.72	77.43	34.28	33.68
F16	Mean	2092.59	2126.74	2140.46	**2040.02**
	Std	162.08	180.25	186.85	195.05
F20	Mean	2224.55	2225.89	2276.96	**2164.04**
	Std	96.75	101.71	94.12	61.11
F26	Mean	**3307.35**	3694.84	3785.91	3443.51
	Std	432.45	595.26	572.65	323.52
F29	Mean	3388.66	**3377.26**	3440.26	3380.73
	Std	101.20	109.16	113.26	88.80

**Table 3:** Intensities at *m*/*z* 172.88 obtained through automatic tuning using different algorithms.

Method	Test 1 (cps)	Test 2 (cps)	Test 3 (cps)	Mean (cps)
Univariate search	2.15 × 10^7^	2.21 × 10^7^	2.16 × 10^7^	2.17 × 10^7^
PSO	2.48 × 10^7^	2.63 × 10^7^	2.64 × 10^7^	2.58 × 10^7^
DE/best/1/bin	2.71 × 10^7^	2.66 × 10^7^	2.70 × 10^7^	2.69 × 10^7^
DE/rand-to-best/1/bin	2.68 × 10^7^	2.64 × 10^7^	2.70 × 10^7^	2.67 × 10^7^
DE-i	2.71 × 10^7^	2.71 × 10^7^	2.73 × 10^7^	2.72 × 10^7^

**Table 4:** Test data of the automatic tuning experiments.

No.	Intensity at *m*/*z* 172.88 (cps)	Intensity at *m*/*z* 622.57 (cps)	Intensity at *m*/*z* 922.36 (cps)
1	2.44 × 10^7^	2.49 × 10^7^	8.29 × 10^6^
2	2.37 × 10^7^	2.31 × 10^7^	7.60 × 10^6^
3	2.56 × 10^7^	2.57 × 10^7^	8.44 × 10^6^
4	2.42 × 10^7^	2.54 × 10^7^	8.41 × 10^6^
5	2.30 × 10^7^	2.53 × 10^7^	7.62 × 10^6^
6	2.73 × 10^7^	2.41 × 10^7^	6.71 × 10^6^
7	2.65 × 10^7^	2.64 × 10^7^	8.23 × 10^6^
8	2.47 × 10^7^	2.63 × 10^7^	8.42 × 10^6^
9	2.53 × 10^7^	2.20 × 10^7^	6.73 × 10^6^
10	2.54 × 10^7^	2.28 × 10^7^	6.79 × 10^6^

## Data Availability

The original contributions presented in the study are included in the article; further inquiries can be directed to the corresponding authors.
